# The Role of GRP78/ATF6/IRE1 and Caspase-12 Signaling Pathways in the Protective Effects of n-Hexane Oil Extract of Black Soldier Flies’ Larvae (*Hermetia illucens*) Against Aflatoxin-Induced Hepatotoxicity in Rats

**DOI:** 10.5812/ijpr-167846

**Published:** 2025-12-28

**Authors:** Fateme Heidari, Tahereh Zarei Taher, Yalda Arast

**Affiliations:** 1 Cellular and Molecular Research Center, Qom University of Medical Sciences, Qom, Iran; 2 Department of Anatomical Sciences, Faculty of Medicine, Ramsar Campus, Mazandaran University of Medical Sciences, Ramsar, Iran; 3 Department of Tissue Engineering and Applied Cell Sciences, Faculty of Medicine, Qom University of Medical Sciences, Qom, Iran; 4 Student Research Committee, Qom University of Medical Sciences, Qom, Iran; 5 Research Center of Environmental Pollutants, Qom University of Medical Sciences, Qom, Iran

**Keywords:** Aflatoxin B1, *Hermetia illucens*, Oxidative Stress, Apoptosis, Liver Injury, GRP78/ATF6/IRE1 Signaling Pathway

## Abstract

**Background:**

Global contamination of agricultural products with aflatoxin (AF) is one of the most important concerns in the field of food safety and quality. Aflatoxin, when entering the food chain, can cause oxidative stress and hepatotoxicity. Black soldier fly larvae (BSFL) are an environmentally friendly insect whose extract is rich in valuable bioactive compounds with antioxidant properties.

**Objectives:**

This study aimed to investigate the protective effect of the n-hexane extract of BSFL on oxidative stress, inflammation, and histopathological changes caused by Aflatoxin B1 (AFB1)-induced hepatotoxicity in rats.

**Methods:**

Thirty-five male Wistar rats with a weight range of 200 to 250 g were randomly divided into 5 equal groups including Control, AFB1 (75 μg/kg), BSFL (360 mg/kg), BSFL 180 mg/kg+AFB1, and BSFL 360 mg/kg+AFB1. At the end of the treatment on the twenty-eighth day, the animals were euthanized and samples were taken for liver enzymes, oxidative stress, inflammation, endoplasmic reticulum (ER) stresses, apoptosis marker, histopathology, and expression of ER stress-related proteins analyses. Data were analyzed by one-way ANOVA followed by Tukey's post-hoc test, with P < 0.05 considered statistically significant.

**Results:**

According to the results of the study, AFB1 administration significantly increased the levels of aspartate aminotransferase (AST), alanine aminotransferase (ALT), alkaline phosphatase (ALP), nitric oxide (NO), malondialdehyde (MDA), tumor necrosis factor alpha (TNF-α), interleukin 1 beta (IL-1β), and interleukin 6 (IL-6) (P < 0.001) and also decreased the levels of superoxide dismutase (SOD), reduced glutathione (GSH), glutathione peroxidase (GPx), and catalase (CAT) compared to the control group (P < 0.001). These biochemical and inflammatory abnormalities caused by AFB1 were confirmed by histopathological observations of liver tissue. Administration of BSFL extract (at the higher dose of 360 mg/kg) resulted in significant reversal of biochemical, inflammatory, and hepatic markers and modulated GRP78/ATF6/IRE1 signaling in AF-poisoned rats. Our histological results showed that BSFL extract (360 mg/kg) could reduce steatosis, cellular swelling, and lobular inflammation induced after AF exposure.

**Conclusions:**

The results of this study indicate that BSFL extract, as a valuable bioactive substance with antioxidant properties, may attenuate biochemical indices, oxidative stress, and inflammation caused by AF-induced hepatotoxicity.

## 1. Background

Mycotoxins are secondary metabolites of filamentous fungi that can contaminate agricultural crops as well as animal products. They pose a threat to the health of humans and animals and can cause irreparable economic losses (1). Mostly, mycotoxins grow in tropical and subtropical regions; however, due to recent climatic and geographical changes, their growth has increased even in temperate areas. According to estimates of the Food and Agriculture Organization (FAO), about 25% of the world’s food production is contaminated with mycotoxins. The most prevalent mycotoxin-producing organisms are *Aspergillus flavus* and *Aspergillus parasiticus*, which produce an important group of mycotoxins known as aflatoxins (AFs). Aflatoxins are heat-resistant, insoluble in water, but exhibit good solubility in organic solvents. Unfortunately, lack of removal of AFs through conventional food processing methods, along with poor post-harvest management, has made food products with long shelf-life such as corn, soybeans, wheat, peanuts, nuts, grains, and dried fruits more susceptible to contamination by this dangerous toxin (1, 2). Another important point regarding the occurrence and spread of AF toxicity is that these toxins can easily be converted into volatile metabolites, which can lead to double contaminations, through contaminating grains consumed by livestock and poultry on farms.

Based on their fluorescence characteristics, AFs are classified into several types, including: Aflatoxin B1 (AFB1), AFB2, AFG1, and AFG2, all of which share a common AF nucleus. Among them, AFB1 exhibits a toxicity level more than ten times higher than the others and is ranked among the most hazardous food contaminants, along with potassium cyanide and arsenic. According to the classification of the International Agency for Research on Cancer (IARC), AFB1 is placed in group A1 (1).

Aflatoxin B1 can easily enter the human food chain through contamination of poultry meat and eggs and can lead to immune system suppression and nutritional disorders, through prolonged exposure. Studies have demonstrated that it is one of the contributing factors in the development of liver, stomach, colorectal, rectal, and gallbladder cancers. Furthermore, exposure to this contaminant is considered one of the most important causes of hepatocellular carcinoma (3).

The liver is considered the primary target organ for AFs and, according to epidemiological studies, one of the most significant risk factors for primary liver cancer. In the liver, AF is metabolized by the cytochrome P450 enzyme system into its carcinogenic form, aflatoxin AFB1-8, 9-epoxide (AFBO). It can also spontaneously convert into the dihydrodiol form, which, in a dose-dependent pattern, causes tissue damage, induces lipid peroxidation and fat accumulation, promotes excessive production of oxygen radicals, triggers apoptosis, enhances inflammatory responses, and stimulates cellular proliferation, all of which contribute to the initiation and development of liver cancer (1, 4).

Black soldier fly larvae (BSFL) oil is rich in bioactive lipids, including unique fats like lauric acid, other beneficial fatty acids, tocopherols (vitamin E), sterols, and phospholipids (5). Lab tests and early animal studies suggest this oil has potent antioxidant and anti-inflammatory effects. The oil extracted using n-hexane from BSFL concentrates these beneficial components, potentially allowing it to target key pathways involved in chemical liver damage such as boosting the body's natural antioxidant defenses [via the nuclear factor erythroid 2-related factor 2 (Nrf2)] and calming harmful inflammation (by suppressing NF-κB) (6, 7). However, a critical gap exists: Despite its promising makeup, no one has tested whether n-hexane BSFL extract specifically protects the liver against a realistic toxin like AFB1. Crucially, its ability to tackle all three damaging aspects of AFB1 toxicity — oxidative stress, inflammation, and physical tissue damage — simultaneously remains unknown. Confirming this potential is vital for establishing BSFL oil as a novel, sustainable therapeutic option. Therefore, this study investigates whether hexane extract from BSFL can protect rats against AF-induced liver damage, specifically examining effects on oxidative stress, inflammation, and liver tissue changes. Therefore, to the best of our knowledge, this is the first study to investigate the protective effects of the n-hexane extract of BSFL against AFB1-induced hepatotoxicity, with a specific focus on elucidating its underlying mechanisms through the modulation of the GRP78/ATF6/IRE1 and caspase-12 signaling pathways, oxidative stress, and inflammation simultaneously.

## 2. Materials and Methods

### 2.1. Ethical Approval

This study was approved by the Research Ethics Committee of Qom University of Medical Sciences (approval code: IR.MUQ.AEC.1403.006). All procedures followed institutional guidelines for animal welfare.

### 2.2. Animals and Housing

Thirty-five male Wistar rats (200 ± 5 g) were obtained from the Animal Studies Center, Qom University of Medical Sciences. Animals were housed under controlled conditions (21 ± 2°C, 12-h light/dark cycle) with free access to standard pellet feed and water. Cages were cleaned daily.

### 2.3. Materials

Black soldier fly larvae oil extract: n-Hexane extract of *Hermetia illucens* larvae was provided under the approved technological proposal of Qom University of Medical Sciences. The n-hexane extract was prepared using the Soxhlet extraction method. The resulting oil was concentrated under reduced pressure using a rotary evaporator, and the solvent was completely removed. The final extract was stored at -20°C until use. The n-hexane solvent was specifically chosen for its high affinity and efficiency in extracting non-polar lipid components. This method is particularly effective for obtaining a high yield of the oil fraction rich in medium-chain fatty acids, such as lauric acid, which is documented as the predominant bioactive compound in BSFL oil, constituting a substantial portion of its fatty acid profile (5, 6, 8). The selection of n-hexane was therefore aimed at maximizing the extraction of these specific lipid-soluble bioactive compounds hypothesized to mediate the hepatoprotective effects.

Chemicals: Aflatoxin B1 (≥ 99%) (Sigma-Aldrich, USA), corn oil (vehicle), formaldehyde, and all assay kits were commercially sourced.

### 2.4. Experimental Design

Rats were randomly assigned to experimental groups using a random number table to minimize bias. After one week of acclimatization, rats were randomly divided into 5 groups (n = 7/group):

Control (corn oil): Received 2 mL/kg/day corn oil orally (9).

Aflatoxin B1 group: Received 75 μg/kg/day AFB1 orally (10).

Black soldier fly larvae group: Received 360 mg/kg/day BSFL extract orally alone (11).

AFB1+BSFL 180 group: Received 75 μg/kg/day AFB1+180 mg/kg/day BSFL extract orally (BSFL administered first, followed within minutes by AFB1).

AFB1+BSFL 360 group: Received 75 μg/kg/day AFB1+360 mg/kg/day BSFL extract orally (BSFL administered first, followed within minutes by AFB1).

Aflatoxin B1 and BSFL extract were dissolved in corn oil prior to administration. The selection of BSFL extract doses (180 and 360 mg/kg) was based on a translational rationale from the known bioactive composition of the extract and existing pharmacological data. Given that lauric acid constitutes the predominant fatty acid (≥ 70%) in BSFL oil (5, 8), the dosing strategy was designed to deliver a therapeutically relevant amount of this key component. The chosen doses correspond to an estimated daily intake of approximately 100 mg/kg and 200 mg/kg of lauric acid, respectively. This range aligns with, and falls within, the effective dosage ranges of lauric acid (100 - 250 mg/kg) previously demonstrated to exert significant hepatoprotective effects in rodent models of toxin-induced liver injury (11). Furthermore, the higher dose (360 mg/kg) was included to evaluate a potential dose-response relationship and ensure a robust test of the extract's efficacy.

Treatments were administered daily for 28 days. Body weights were recorded at baseline and on day 29.

### 2.5. Sample Collection

On day 29, rats were anesthetized using ketamine (50 mg/kg) and xylazine (10 mg/kg) (12). Blood was drawn via cardiac puncture into sterile tubes. Serum was separated by centrifugation (4000 × g, 10 min, 4°C) and stored at -20°C for liver function analysis. Liver tissues were rapidly excised. Sections were fixed in 10% neutral buffered formalin for ≥ 24 h for histopathology. Remaining tissue was homogenized (1:10 w/v) in ice-cold Tris-HCl buffer (0.1 M, pH 7.4), centrifuged (12,000 × g, 15 min, 4°C), and the supernatant stored at -80°C for biochemical assays (13).

### 2.6. Biochemical Assays

Liver function markers (serum): Alanine aminotransferase (ALT), aspartate aminotransferase (AST), and alkaline phosphatase (ALP) levels were measured using commercial colorimetric kits (Wiesbaden, Germany) using a spectrophotometer (UNICO Instruments C., Model 1200, USA).

Oxidative stress markers (liver homogenate): Lipid peroxidation: The malondialdehyde (MDA) level was evaluated using the MDA assay kit according to the protocol of the company [Teb Pazhouhan Razi (TPR), Tehran, IRAN]. Liver nitric oxide (NO) levels were measured using the Griess diazotization reaction after conversion of nitrate to nitrite by nitrate reductase in supernatant (Nitric Oxide Assay Kit, Navand Lab Kit, Tehran, IRAN). Antioxidant enzymes: The catalase (CAT) enzyme activity was measured by kit (Catalase Activity Assay Kit, Navand Lab Kit, Tehran, IRAN). Superoxide dismutase (SOD) activity was evaluated using the SOD assay kit according to the protocol of the company (SOD Activity Assay Kit, Navand Lab Kit, Tehran, IRAN). The method described by Ellman (Ellman 1959) was used for glutathione S-transferase (GST) analysis [GSH Activity Assay Kit, Navand Lab Kit, Tehran, IRAN]. Glutathione peroxidase (GPx) activity was measured with the GSH peroxidase kit (GPx Activity Assay Kit, Navand Lab Kit, Tehran, IRAN). The total protein content of the liver tissue homogenate sample was determined with the method developed by Bradford (14).

Inflammatory markers (liver homogenate): The levels of hepatic inflammatory factors, including tumor necrosis factor alpha (TNF-α), interleukin 1 beta (IL-1β), and interleukin 6 (IL-6), were determined by ELISA using the relevant kits (CN: KPG-TNF-α -48, CN: KPG-RIL1β, CN: KPG-RIL6, Karmania Pars Gene, Tehran, Iran) and IL-1β Assay Kit (IBL Company, code No. 27193), and the results were expressed as pg./mg of protein.

### 2.7. Western Blot Assay

Western blot analyses were performed as previously described with some modifications (15, 16). For western blotting, tissue was lysed with RIPA buffer. The lysates were removed by centrifugation at 14,000 rpm for 20 min at 4°C. Protein concentration was determined by the Bradford Protein Quantification Kit (DB0017, DNAbioTech, Iran) according to the manufacturer's instructions. The tissue lysates were mixed with equal volume of 2X Laemmli sample buffer. Lysates (20 μg) were then subjected to SDS-PAGE after a 5 min boiling and subsequently transferred to a 0.2 μm Immune-Blot™ polyvinylidene difluoride (PVDF) membrane (Cat No: 162-017777; Bio-Rad Laboratories, CA, USA). The membranes were then blocked with 5% BSA (Cat No: A-7888; Sigma Aldrich, MO, USA) in 0.1% Tween 20 for 1 h. Then, the membranes were incubated with anti-β actin-loading control antibodies (1/2500, Cat No: ab8227, Abcam) for 1 h at room temperature. Subsequently, membranes were washed thrice with TBST and incubated with goat anti-rabbit IgG H&L (HRP) (1/10000, Cat No: ab6721; Abcam) secondary antibody. The membranes were then incubated with enhanced chemiluminescence (ECL) for 1 - 2 min. Protein expression was normalized to β-actin. Densitometry of protein bands was performed by an investigator blinded to the groups using the gel analyzer Version 2010a software (NIH, USA), such that the percentage area under the curve of each band was divided by the percentage area under the curve of its corresponding actin band, and then calculated values were compared between groups as we described previously (17).

### 2.8. Tissue Preparation and Histopathological Examination

Liver samples were fixed in 10% neutral buffered formalin, dehydrated through graded ethanol, cleared in xylene, and embedded in paraffin. Sections of 5 µm thickness were mounted on glass slides and stained with hematoxylin and eosin (H&E) following Bancroft and Layton (18). Masson’s Trichrome staining was used for subjectively evaluating the amount and the distribution of mature collagen fibers in liver tissue and indicated the fibrosis in liver. Histological evaluation and imaging were performed using a light microscope (Leica DM750, Leica Microsystems, India). An experienced pathologist blinded to the experimental groups interpreted all slides. Tissue alterations were quantitatively analyzed using Image software (NIH, Bethesda, MD, USA).

### 2.9. Statistical Analysis

Data are expressed as mean ± standard deviation(SD). Differences were analyzed by one-way ANOVA followed by Tukey's post-hoc test using SPSS/GraphPad Prism. P < 0.05 was considered significant. A power calculation was not performed a priori; the sample size was determined based on common practices in similar toxicological studies. This is acknowledged as a limitation in the discussion section.

## 3. Results

### 3.1. Black Soldier Fly Larvae Extract Effect on Hepatic Markers in the Aflatoxin-Induced Model

**Figure 1.**
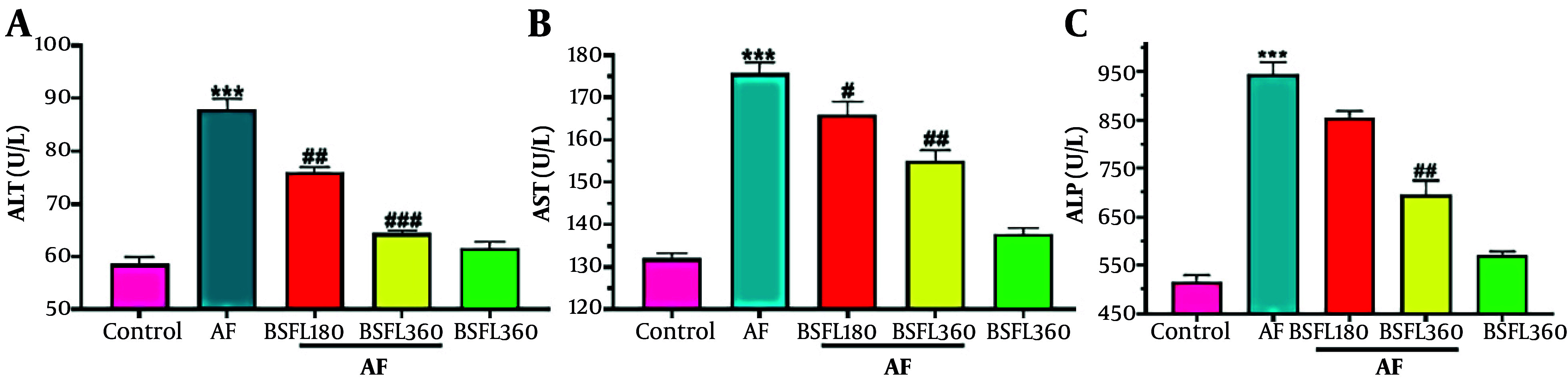
Treatment with black soldier fly larvae (BSFL) at two different concentrations (180 and 360 mg/kg) on alanine aminotransferase (ALT)(A), aspartate aminotransferase (AST)(B), and alkaline phosphatase (ALP)(C) levels in the aflatoxin-induced model (AF-induced model). Values are expressed as mean ± standard deviation (SD); n = 7. * Significant difference in comparison with the control group (*** P < 0.001). # Significant difference in comparison with the AF group (# P < 0.05; ## P < 0.01; ###P < 0.001).

Figure 1A-C shows the biomarkers of hepatic function in rats treated with AFB1 or BSFL extract alone and in combination. Hepatic ALT activity in serum was increased (P < 0.001) resulting from AFB1 administration alone compared to the control group. The increase in this biomarker of hepatotoxicity was reduced significantly in co-treated rats with AFB1 and BSFL in 180 and 360 mg/kg concentrations (P < 0.01 and P < 0.001; Figure 1A). Hepatic AST levels increased significantly with AFB1 treatment (P < 0.001), while BSFL in 180 and 360 mg/kg decreased AST activity (P < 0.05 and P < 0.001; Figure 1B). Changes in the level of ALP also increased significantly after administration of AFB1 alone, similar to the pattern of ALT and AST, compared to the control group. The increase in the level of ALP due to AFB1 was significantly reduced after treatment with the BSFL at 180 and 360 mg/kg. This reduction was significantly greater at 360 mg/kg and close to the control group (P < 0.01; Figure 1C). Nitric oxide significant difference in ALT, AST, and ALP levels was observed in the group that received BSFL at 360 mg/kg alone compared to the control group.

### 3.2. Black Soldier Fly Larvae Extract Effect on Hepatic Antioxidant Status and Nitrative Stress in the Aflatoxin-Induced Model

**Figure 2.**
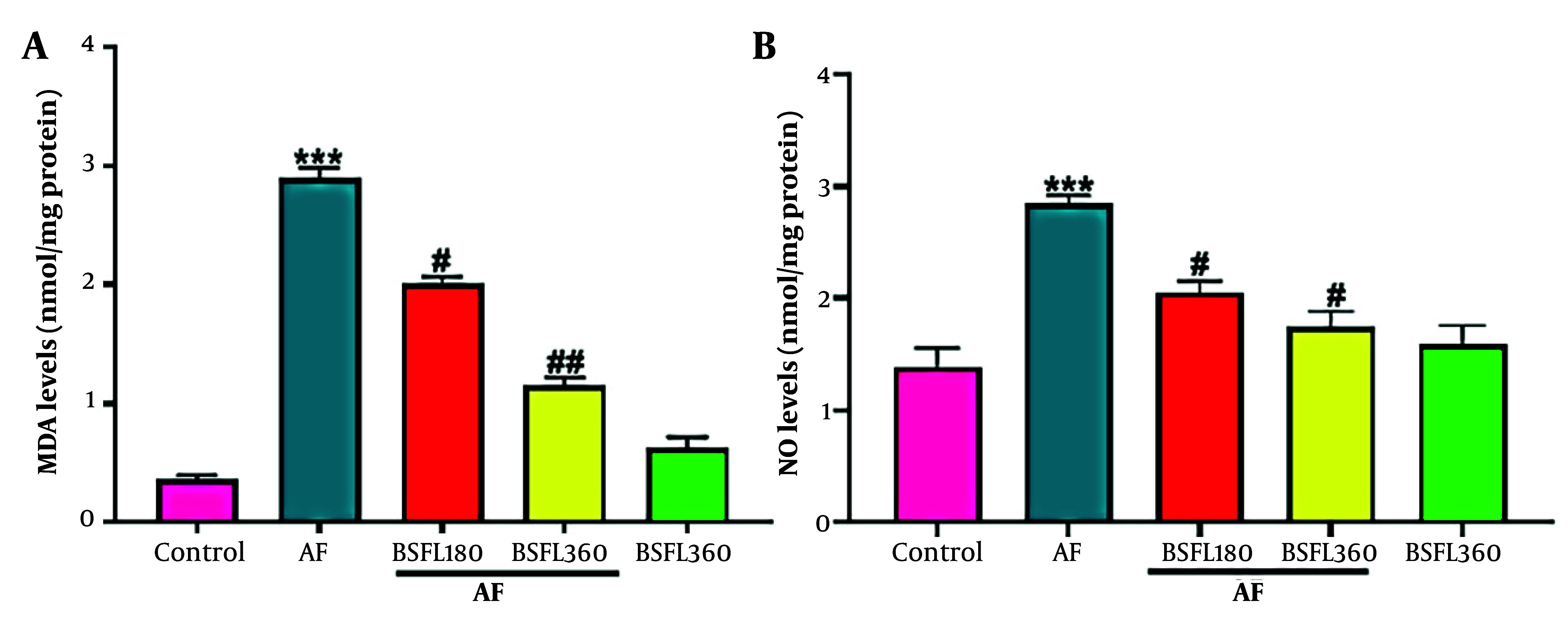
Treatment with black soldier fly larvae (BSFL) at two different concentrations (180 and 360 mg/kg) on malondialdehyde (MDA)(A) and nitric oxide (NO)(B) levels in the aflatoxin-induced model (AF-induced model). Values are expressed as mean ± standard deviation (SD); n = 7. * Significant difference in comparison with the control group (*** P < 0.001); # Significant difference in comparison with the AF group (# P < 0.05 and ## P < 0.01)

Figure 2A-B shows the liver oxidative stress parameters. Compared to the control group, in the AFB1 group, MDA levels significantly increased (P < 0.001), but after administration of BSFL at 180 and 360 mg/kg concentrations, the MDA level decreased (P < 0.05 and P < 0.001 respectively; Figure 2A). Aflatoxin B1 application significantly increased NO levels compared to the control group (P < 0.001), but in co-treated rats with AFB1 and BSFL (180 and 360 mg/kg), NO levels decreased significantly (P < 0.05, respectively; Figure 2B). Furthermore, no significant changes in MDA and NO levels were observed in the group that received BSFL at 360 mg/kg alone compared to the control group.

**Figure 3.**
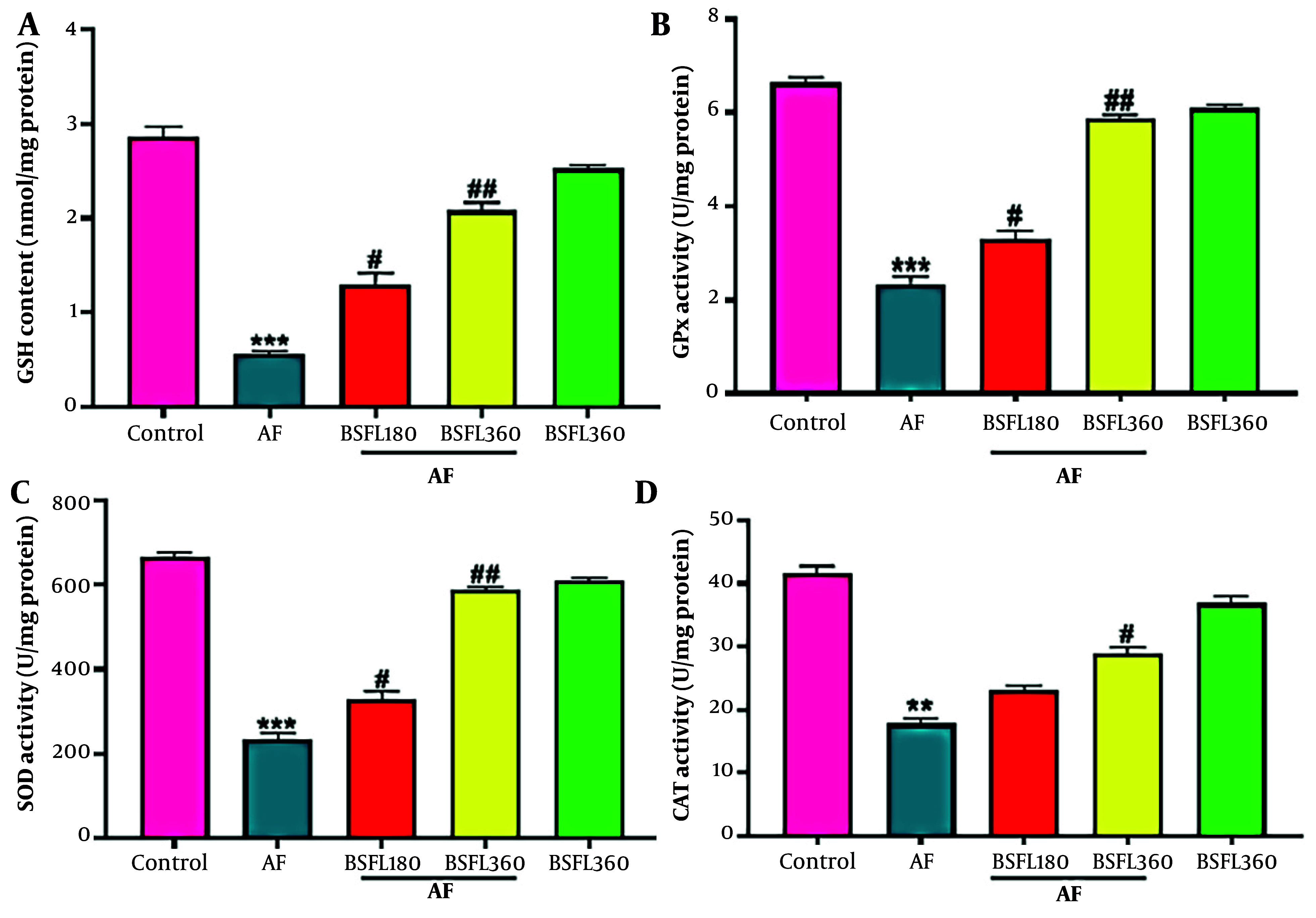
Treatment with black soldier fly larvae (BSFL) at two different concentrations (180 and 360 mg/kg) on reduced glutathione (GSH)(A) content and glutathione peroxidase (GPx)(B), superoxide dismutase (SOD)(C), and catalase (CAT)(D) activity in the aflatoxin-induced model (AF-induced model). Values are expressed as mean ± (SD); n = 7. * Significant difference in comparison with the control group (** P < 0.01 and *** P < 0.001); # Significant difference in comparison with the AF group (# P < 0.05 and ## P < 0.01)

Figure 3A-D shows the liver antioxidant levels. Reduced glutathione contents were statistically significantly different (P < 0.001) between AFB1 and control groups, and when BSFL at 180 and 360 mg/kg concentrations was administered along with AFB1, GSH contents significantly increased (P < 0.05 and P < 0.01, respectively; Figure 3A). Aflatoxin B1 administration alone demonstrated a significant decrease in GPx activity compared to the control group (P < 0.001); however, when BSFL at 180 and 360 mg/kg concentrations was administered with AFB1, an increase in GPx activity was observed (P < 0.05 and P < 0.001, respectively; Figure 3B). A statistically significant decrease was observed in SOD and CAT activity between AFB1 and control group (P < 0.001 and P < 0.01, respectively), and application of BSFL at 360 mg/kg with AFB1 significantly increased the SOD and CAT activity (P < 0.001 and P < 0.05 respectively). Black soldier fly larvae at 180 mg/kg with AFB1 increased SOD level (P < 0.05) while it did not significantly increase the CAT at the same concentration (Figure 3C-D). Nitric oxide significant changes in GSH, GPx, SOD, and CAT levels were observed in the group that received BSFL at 360 mg/kg alone compared to the control group.

### 3.3. Black Soldier Fly Larvae Extract Effect on Liver Pro-Inflammatory Cytokines in Aflatoxin-Induced Model

**Figure 4.**
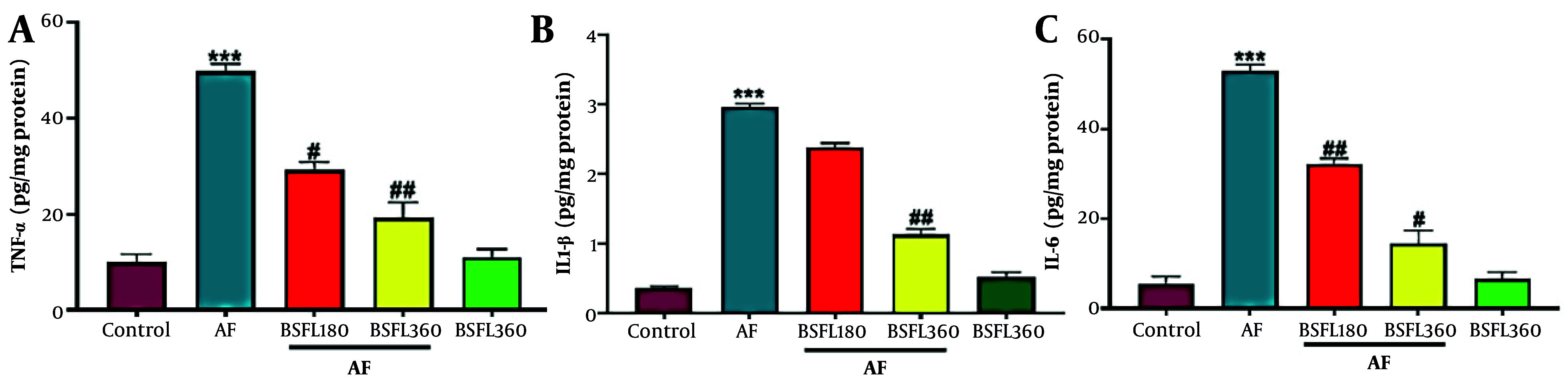
Treatment with black soldier fly larvae (BSFL) at two different concentrations (180 and 360 mg/kg) on tumor necrosis factor alpha (TNF-α)(A), interleukin 1 beta (IL-1β)(B), and interleukin 6 (IL-6)(C) in the aflatoxin-induced model (AF-induced model). Values are expressed as mean ± standard deviation (SD); n = 7. * Significant difference in comparison with the control group (*** P < 0.001); # Significant difference in comparison with the AF group (# P < 0.05 and ## P < 0.01)

Figure 4A-C show the effect of AFB1 and BSFL treatment on inflammatory mediators. Administration of AFB1 alone increased hepatic cytokines: Tumor necrosis factor alpha, IL-1β, and IL-6 compared to control group (P < 0.001). In the treatment by BSFL at 180 mg/kg concentration, the levels of TNF-α and IL-6 significantly reduced compared to the control group (P < 0.05 and P < 0.001, respectively; Figure 4A, C). Nitric oxide significant change was observed at this concentration for IL-1β (Figure 4B). Treatment by BSFL at 360 mg/kg concentration significantly reduced levels of TNF-α, IL-1β, and IL-6 in the aflatoxin-induced model (AF-induced model; P < 0.01, P < 0.01, and P < 0.05, respectively). Nitric oxide significant changes in TNF-α, IL-1β, and IL-6 levels were observed in the group that received BSFL at 360 mg/kg alone compared to the control group.

### 3.4. Black Soldier Fly Larvae Extract Effect on Liver Histopathological Damages in Aflatoxin-Induced Model

**Figure 5.**
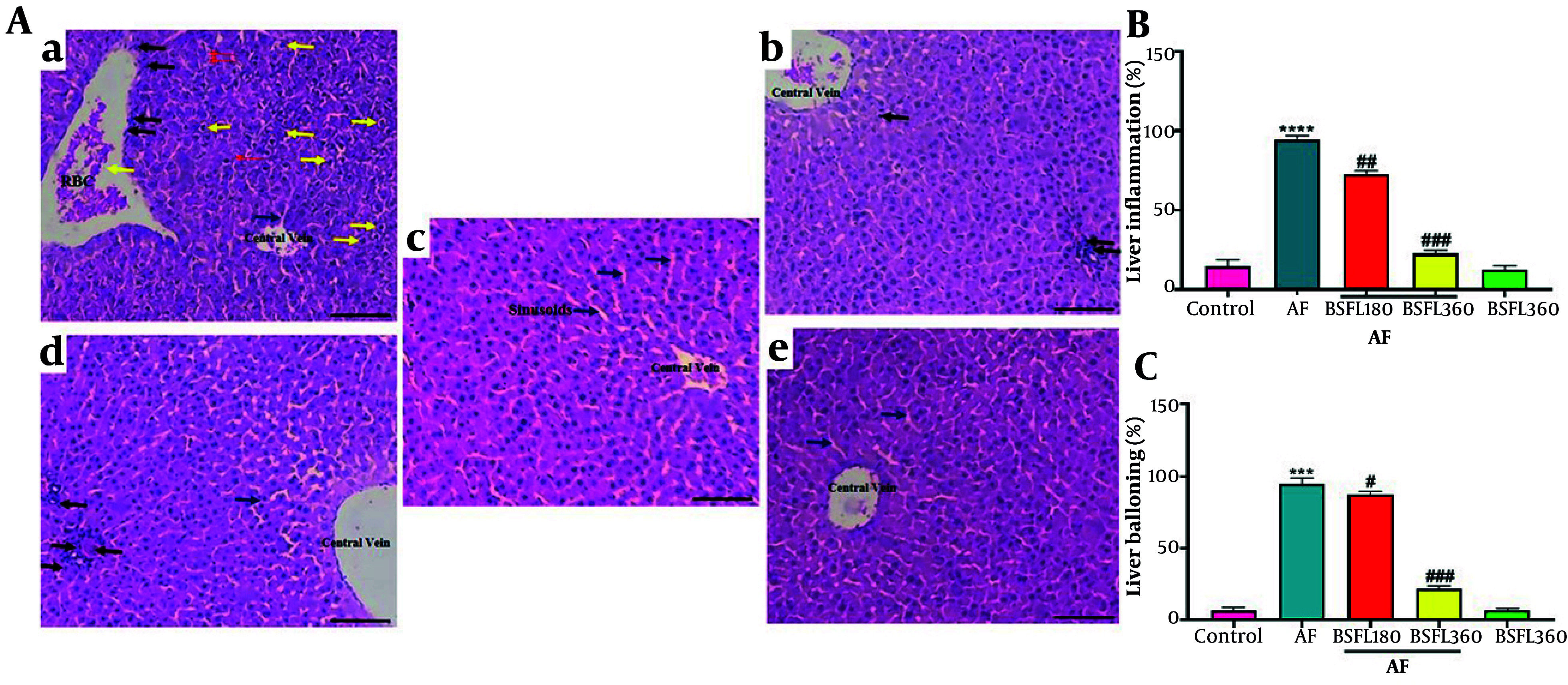
Histological evaluation of liver tissues after hematoxylin and eosin (H & E) staining (magnification 100X) and quantitative analysis of injury parameters.A, reperesentative micrographs: a, AFB1 group, b, BSFL 360 alone, c, Control, d, AFB1 + BSFL 180, e, AFB1 + BSFL 360. Yelow arrow: Balloooning degeneration; blue arrow: Sinusoidal space; red arrow: RBCs; black arrow: Inflammatory infiltration. B, quatitative analysis of lobular inflammation (%). C, quantitative analysis of hepatocyte ballooning (%). Data are expressed as mean ± SD, n = 7; * Significant difference compared with the control group (*** P < 0.001 and **** P < 0.0001). # Significant difference compared with the AFB1 group. ( # P < 0.05, ## P < 0.01, ### P < 0.001).

Histological results in Figure 5 showed severe steatosis (excessive lipid droplet accumulation in hepatocytes), cellular ballooning, and lobular inflammation after AF challenge. Administration of BSFL (360 mg/kg) prevented AFB1-induced lipid accumulation, cellular ballooning, and lobular inflammation. However, administration of a low dose of BSFL (180 mg/kg) could not improve liver steatosis and inflammation in the AFB1-induced rats.

**Figure 6.**
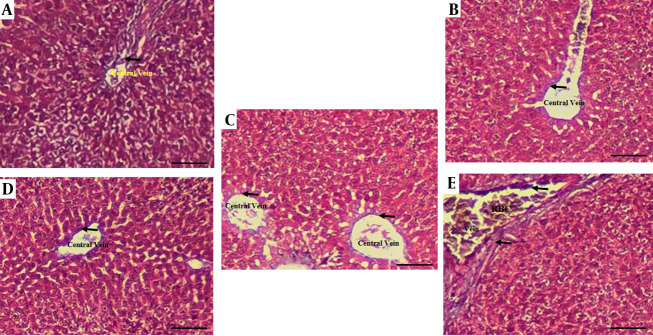
Histological findings of liver tissues after Masson’s Trichrome staining (magnification 100X) in different experimental groups. A, aflatoxin-induced model (AF-induced model), B, black soldier fly larvae (BSFL) 360 (BSFL at 360 concentration); C; control: normal liver architecture; D, AF+BSFL 180 (AF-induced model treated with BSFL at 180 mg/kg concentration); E, AF+BSFL 360 (AF-induced model treated with BSFL at 360 mg/kg concentration); black arrows show collagen fibers.

The histological assessment of liver fibrosis via Masson's Trichrome staining revealed distinct morphological changes across the experimental groups, as summarized in Figure 6. The AF group exhibited a marked deposition of collagen fibers, indicated by black arrows, confirming the onset of hepatic fibrosis. In contrast, livers from the control group maintained normal architectural integrity with minimal collagen presence. Therapeutic intervention with BSFL extract demonstrated a dose-dependent ameliorative effect. The group treated with the lower dose (AF+BSFL 180) showed a noticeable reduction in collagen accumulation compared to the AF group. Importantly, the group receiving the higher dose (AF+BSFL 360) presented a histo-architecture that closely resembled the control group, with a significant decrease in fibrous deposits. These findings suggest that BSFL extract possesses potent anti-fibrotic properties, effectively mitigating AF-induced collagen deposition in the liver.

### 3.5. Black Soldier Fly Larvae Extract Effect on Endoplasmic Reticulum Stress-Related Molecules in Aflatoxin-Induced Model - Analysis of Key Molecular Markers by Western Blot

**Figure 7.**
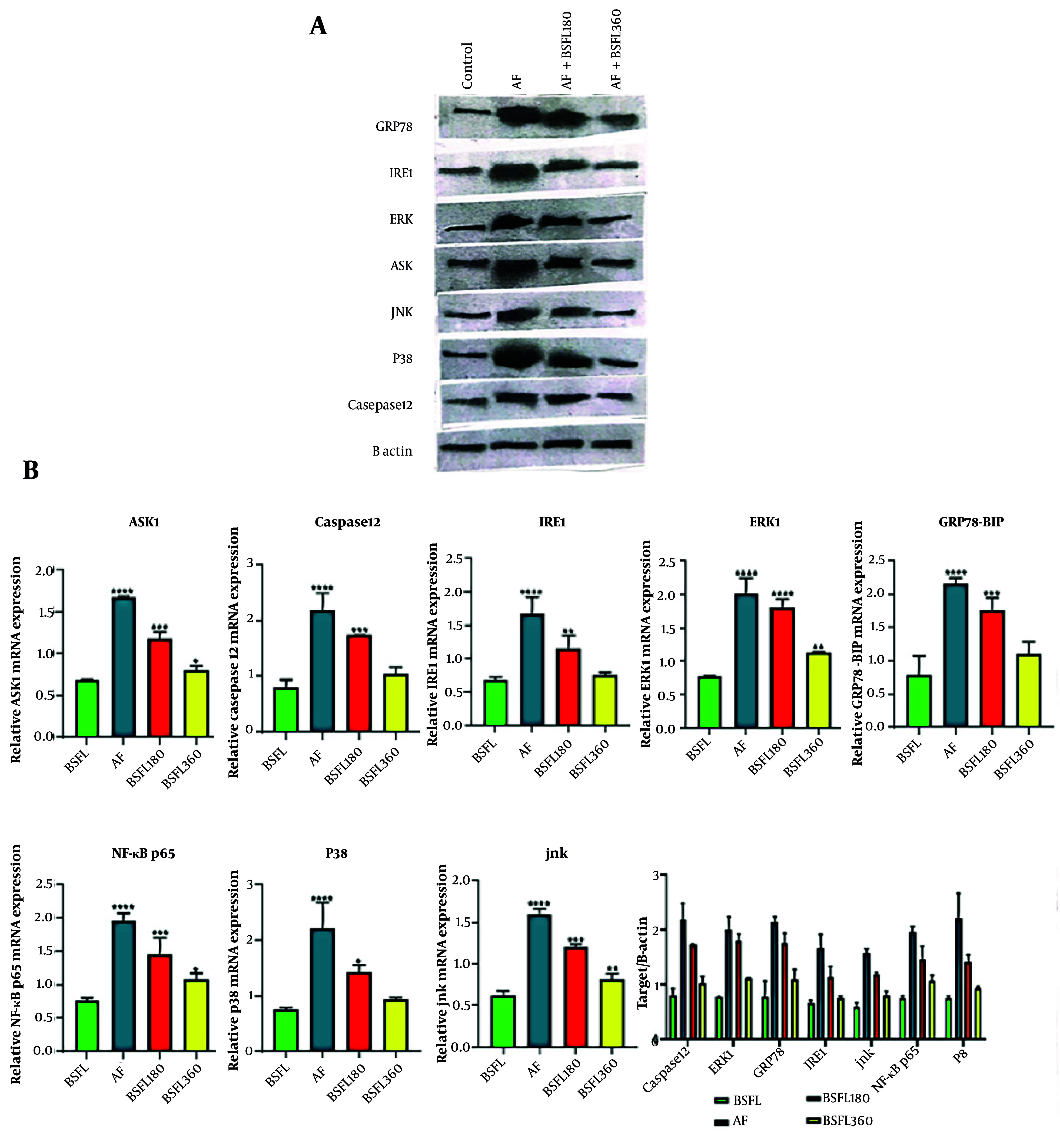
Tratmen with Black Soldier Fly Larvae at two different concentration (180 and 360 mg/Kg) on endoplasmic reticulum stress and apoptosis-related signaling pathway in the aflatoxin- induced model. A, reperesentative Western blot images shoewing protein expression levels of ASK1, caspase-12, IRE1, ERK1, GRP78, NF-κB, P38, and JNK in liver tissue. B actin was used as a loading control. B, densitometry quantification of protein expression normalized to B actin : ASK1, Caspase 12, IRE1, ERK1, GRP78, NF-KB, p38, JNK. Data expressed as means ± SD (n = 7).* Significant difference compared with the cntrol(BSFL) group (* P < 0.05, ** P < 0.01, *** P < 0.001 and **** P < 0.0001).

Western blot analysis was performed to elucidate the molecular mechanisms underlying the hepatoprotective effects of BSFL extract against AFB1-induced toxicity (Figure 7A). The quantitative results, normalized to the loading control and presented in Figure 7B, revealed significant alterations across the experimental groups. The AFB1-intoxicated group exhibited a pronounced upregulation of pro-apoptotic and pro-inflammatory signaling molecules compared to the normal control group. Particularly: The expression of JNK-phosphate and apoptosis signal-regulating kinase 1 (ASK1) increased in the AFB1 group, indicating a strong activation of stress kinase pathways. Endoplasmic reticulum (ER) stress marker GRP78/BiP was upregulated. The inflammatory transcription factor NF-κB and the key apoptotic executioner, caspase 12, were elevated.

The AFB1-intoxicated group exhibited a pronounced upregulation of pro-apoptotic and pro-inflammatory signaling molecules compared to the normal control group (M). Endoplasmic reticulum stress and apoptosis: Apoptosis signal-regulating kinase 1 and its downstream target, GRP78 (a key ER stress chaperone), were markedly elevated in the AFB1 group. This indicates the induction of ER stress-mediated apoptosis. The expression of the central inflammatory transcription factor NF-κB was significantly higher in the AFB1 group, confirming the activation of inflammatory pathways. Stress kinase pathways: Key members of the mitogen-activated protein kinase (MAPK) family, which are responsive to cellular stress, were activated. This was evidenced by increased levels of phosphorylated JNK and p38. Apoptosis execution: The increase in caspase levels in the AFB1 group points towards the activation of the executive phase of apoptosis. Nevertheless, the expression of ERK1, a kinase often associated with cell survival and proliferation, was downregulated in the AFB1 group, further tilting the balance towards cell death.

Treatment with the BSFL extract, particularly at the higher dose (360 mg/kg), demonstrated a remarkable ability to counteract the detrimental effects of AFB1. Co-treatment with the extract (180 and 360 mg/kg groups) resulted in a dose-dependent attenuation of the protein levels elevated by AFB1. The expression of ASK1, GRP78, NF-κB, JNK, p38, and caspase was significantly reduced in the BSFL (180 and 360 mg/kg) groups compared to the AFB1 group. Furthermore, the BSFL extract treatment contributed to restoring the level of pro-survival ERK1 towards normal values.

These findings suggest that the protective effect of the BSFL extract is mediated through the modulation of critical signaling pathways. The suppression of ASK1 and GRP78 indicates an alleviation of ER stress. The downregulation of NF-κB signifies a potent anti-inflammatory activity, likely reducing the production of pro-inflammatory cytokines. The inhibition of the JNK and p38 pathways, along with the reduction in caspase, points to a strong anti-apoptotic effect, preventing hepatocyte cell death (Figure 7B). Rather, the expression of ERK1, a kinase associated with cell survival, was significantly downregulated in the AFB1 group — treatment with the BSFL extract demonstrated a remarkable, dose-dependent attenuation of the protein levels elevated by AFB1. Treatment with the lower dose of BSFL extract (180 mg/kg) significantly reduced the expression of all elevated markers. For instance, JNK-phosphate and ASK1 levels were brought down compared to the control group. The higher dose of the extract (360 mg/kg) showed a dose-dependent manner, normalizing the expression of most proteins. The levels of JNK-phosphate, ASK1, GRP78/BiP, and NF-κB were reduced to near baseline levels. Notably, the expression of the pro-survival protein ERK1 was also restored to normal levels with the high-dose treatment.

## 4. Discussion

Aflatoxin B1 is a highly toxic and hazardous mycotoxin which is produced as a secondary metabolite by filamentous fungi. First of all, AFB1 contaminates agricultural products and subsequently enters the human food chain through animal-derived products such as milk, eggs, and meat, hence posing a serious threat to human health (1). Toxicological studies have demonstrated that AF can disrupt the function of various organs, including the heart, brain, hepatic, and renal systems after entering the human body. However, based on epidemiological evidence, the liver is the primary target organ for AF toxicity (10). Prolonged exposure to AF, which is considered as a natural toxin, can also exert toxic effects on the nervous and immune systems, and have teratogenic and genotoxic effects. Cellular toxicity studies have demonstrated that exposure to AF, in a dose-dependent manner, can induce its cytotoxic effects through mechanisms such as cell cycle arrest, apoptosis induction, and oxidative stress. Albeit, the manifestation of these cytotoxic effects is strongly influenced by factors such as the exposure dose, route of exposure, presence of other toxic agents, and the specific characteristics of the target organ (10).

The black soldier fly is an environmentally friendly insect that is able to convert organic waste into valuable bioactive compounds and materials. Nowadays, with the rapid growth of the global population and increasing concerns regarding the supply of animal protein, using insects is recognized as a promising alternative protein source with unique nutritional value (19). On the other hand, in previous studies, it has been demonstrated that oil extracts derived from BSFL possess antioxidant properties. Today, BSFL are mainly used in animal, poultry, and aquaculture feed. However, given their valuable fatty acid and amino acid profile, they can be used through proper processing and the development of a scientific formulation to serve in the future as a suitable alternative to compounds whose production and supply currently require very high costs.

A comparison of the fatty acid profile of BSFL with natural fatty acid sources such as coconut oil, palm oil, and olive oil has shown that coconut oil contains about 45 - 53% lauric acid, and palm oil contains approximately 48% of this 12-carbon fatty acid, whereas the lauric acid content in larval oil has been reported to exceed 70% (8). However, it is worthy of mention that the production of oil from BSFL is far more cost-effective and efficient, compared with plant-based fatty acid sources, and it is due to the larvae’s shorter production cycle and their ability to feed on low-value organic waste. This not only contributes to a sustainable economy but also supports key public health objectives within the community (8, 20).

Previous studies have demonstrated that BSFL can be effective in reducing hepatic fat synthesis and concentration, as well as improving metabolic health parameters — effects that can largely be attributed to lauric acid, which constitutes a substantial portion of the fatty acids present in the extract (8). The significant reduction in serum hepatic enzymes by BSFL extract not only confirms its hepatoprotective property but also suggests a potential mechanism shared with lauric acid, its predominant component. While our findings align with the study by Namachivayam and Gopalakrishnan on lauric acid (11), it is noteworthy that the whole BSFL extract exhibited effects at doses that deliver a lower equivalent amount of lauric acid. This discrepancy may indicate a synergistic contribution from other bioactive lipids or compounds in the extract, amplifying the protective effect beyond that of lauric acid alone. Future studies isolating individual components are warranted to test this hypothesis. Namachivayam and Gopalakrishnan also reported similar results about hepatic enzymes after the administration of lauric acid.

Oxidative stress is probably one of the most important factors contributing to cellular toxicity. The production of reactive oxygen and nitrogen, along with lipid oxidation in cell membranes and the disruption of membrane integrity, will result in an imbalance between intracellular oxidant and antioxidant systems, finally weakening the cell’s antioxidant defense mechanisms (11). In the present study, administration of AF led to an increase in NO levels as well as MDA, the end product of lipid peroxidation, which is totally in line with the previously described mechanisms of AF-induced cytotoxicity. Additionally, administration of AF, so as to counteract the body’s antioxidant defense system, caused a significant decrease in hepatic enzyme levels. However, treatment with the extract at both effective concentrations reduced NO and MDA levels, while enhancing the activity of antioxidant defense markers in the liver and restoring them toward the levels observed in the control group.

This effect may indicate the presence of compounds in the BSFL extract that activate NO synthase. While excessive NO can be harmful, the lack of toxicity in the extract-only group is indicative of a beneficial and possibly regulated increase in NO-dependent processes, such as vasodilation and cellular protection, which may contribute to the overall hepatoprotective effect.

Inflammation is also one of the key factors in the progression of chronic diseases, particularly liver disorders. In this study, administration of AF increased pro-inflammatory factors and cytokines such as IL-1β, IL-6, and TNF-α. The increased secretion of these inflammatory cytokines not only contributed to secondary hepatic tissue damage but also intensified the primary injury (21). However, in the groups treated with BSFL extract, inflammation was significantly reduced in a dose-dependent manner. Based on the results of Western blot in the present study, the reduction of inflammatory factors after administration of the BSFL extract may be linked to the extract’s inhibitory effect on NF-κB signaling, which plays an important role in regulating inflammatory responses in hepatic tissue.

Moreover, studies on BSFL extract have demonstrated that by inducing oxidative stress and activating the caspase cascade — an upstream event in apoptosis — in a dose-dependent manner, it can affect the induction of cancer cell death in melanoma cell lines (22).

As previously mentioned, various mechanisms have been reported in studies regarding AF-induced cytotoxicity. The ER is considered one of the main intracellular organelles targeted by AF toxicity and is highly sensitive to external stimuli. Through the production of free radicals, AF can attack the ER leading to inflammation and apoptosis, which finally cause damage and dysfunction (23). Whenever the ER undergoes stress, the levels of unfolded or misfolded proteins within the cell increase. Thus, the ATF6 proteins dissociate from GRP78, so that they can combine with the unfolded proteins (24). According to studies, many natural compounds can reduce ER stress (25). The increased level of phosphorylated JNK in the AF group indicates the fundamental role of oxidative stress in signaling and the progression of apoptosis. Apoptosis signal-regulating kinase 1 is a redox-sensitive kinase that activates both the JNK and p38 pathways, finally leading to cell death (26). The simultaneous increase of GRP78 and caspase-12 further confirms the key role of ER stress in promoting apoptosis (24, 27).

The Western blot results in this study support the presence of complex underlying intracellular signaling mechanisms involved in hepatoprotection. The increased expression of GRP78, as a key marker of ER stress, and its upstream regulator ASK1 in the AF group is indicative of the fact that AF plays an important role in triggering apoptosis in hepatic cells by inducing ER stress. However, according to the findings of Obsilova et al., inhibition of ASK1 under stress conditions can exert a hepatoprotective effect (28). The present study demonstrated that the BSFL extract effectively suppressed ER stress by reducing the expression of ASK1 and GRP78, which is totally in line with the findings of the previous study.

In this study, the BSFL extract also reduced the activation of JNK and p38 MAP kinase pathways, which are stress-sensitive kinases and are effective in the induction of inflammation. In the study by Schuster-Gaul et al., inhibition of ASK1 was shown to exert hepatoprotective and anti-inflammatory effects by activating the NLRP3 pathway, leading to decreased hepatocyte death and fibrosis resulting from NLRP3-mediated inflammatory signaling (29). Additionally, both p38 and JNK can reduce the expression of fibrogenic genes by phosphorylating nuclear transcription factors such as ATF6 and c-Jun. The reduced expression levels of p38 and JNK genes and proteins indicate a protective effect against hepatic ischemic injury.

Simultaneous administration of the BSFL extract was also able to modulate the level of ERK1, a kinase associated with cell survival and proliferation. Transferring the balance from pro-apoptotic signaling pathways such as p38 and JNK to pro-survival ERK1 pathways shows a vital mechanism in maintaining hepatocyte integrity (30, 31). The BSFL extract demonstrated a significant role in promoting hepatocyte survival and protection following AF-induced toxicity, through constructive intermediation of this mechanism.

Our histological results showed severe steatosis (excessive lipid droplet accumulation in hepatocytes), cellular ballooning, and lobular inflammation after AF challenge, based on many previously published studies (32, 33); but, 360 mg/kg BSFL supplementation prevented AFB1-induced lipid accumulation, cellular ballooning, and lobular inflammation. However, administration of a low dose of BSFL (180 mg/kg) could not improve liver steatosis and inflammation in the AFB1-induced rats. The histological results were compatible with those obtained from expression of genes related to AF and confirmed the positive effect of BSFL in preventing AFB1-induced liver injuries.

While this study provides compelling evidence for the hepatoprotective effects of BSFL extract, it is important to critically appraise its findings within their context. A key limitation is the use of a whole extract, which, while ecologically relevant, makes it challenging to attribute the observed effects solely to lauric acid despite its predominance. The efficacy of our extract at doses delivering a lower equivalent of lauric acid compared to some pure compound studies (11) suggests possible synergism with other medium-chain fatty acids or minor constituents, a hypothesis requiring validation through compound isolation studies. Furthermore, the focus on a single extraction solvent (n-hexane) may have selected for a specific lipid profile; testing extracts from other solvents could reveal additional bioactive spectra. Finally, while the acute AFB1 model is well-established, the therapeutic potential of BSFL extract needs evaluation in chronic, low-dose exposure scenarios, which more accurately mimic human dietary exposure, and in other species to assess translatability.

In conclusion, our findings suggest that the n-hexane oil extract of BSFL may confer a protective effect against AF-induced hepatotoxicity, potentially through a synergistic combination of mechanisms. It functions as a potent antioxidant, directly and indirectly bolstering the cellular defense system against reactive oxygen species (ROS). It acts as an effective anti-inflammatory agent by inhibiting the NF-κB pathway. Moreover, it modulates critical cell signaling pathways, alleviating ER stress and shifting the balance from apoptosis towards cell survival. The consistent dose-dependent efficacy and the absence of intrinsic toxicity point to the therapeutic potential of BSFL extract as a natural protective agent against chemical-induced liver injury. Future studies should focus on identifying the specific bioactive compounds responsible for these effects and elucidating their precise molecular targets, particularly their interaction with the Nrf2 and NF-κB pathways.

## Data Availability

The dataset presented in the study is available on request from the corresponding author during submission or after publication.
